# Supplementary material for: Chromothripsis during telomere crisis is independent of NHEJ, and consistent with a replicative origin
Source: Genome Res. 2019 May;29(5):737–49. doi: 10.1101/gr.240705.118 (PMC6499312; doi:10.1101/gr.240705.118)
Supplement: Supplemental Material [file supp_gr.240705.118_Supplemental_file_1.zip › contigs/annotated_contigs/DB38/contig.2.DB38_length_604_mean_cov_8.41225165563.docx]

**DB38_length_604_mean_cov_8.41225165563**

```
TGTTATATGGCAAGAGATAGGAGGCTAGTTTCATTCTTCTGCATAGGGATATCTAGTTTTCCCACCACCACTTATTGAAGAAACTGTCC
 >chr6:126712788-126713062 + E=3e-153 p=4e-02
TTTCCCCAGTGTATGTTTTTGGCACCTTTGTAAAAAATGAGTTCACTGTAGATGTATGGATTTATTTCTGGGTTCTCTGTTTTGTTCCA

CTGGCCCATATGTCTGTTTTTATGCCAGTGCCATGCTGTTTTGGGTACTATAGCTGTGTAGCATCATTTGAAATCAGGTAATGTGATTC

CTCCA|GT|ATATGCACCCAATACAGGAGCACCCAGATTCATAAAGCAAGTCCTGAGTGACCTACAAAGAGACTTAGGCTCCCACACAT
      >chr6:126997023-126997352 + E=3e-177
TAATAATGGGAGACTTTAACACCCCAATGTCAAAATTAGACAGATCAACAAGACAGAAAGTCAACAAGGATACCCAGGATTTGTACTCA

GCTCTGCACCAAGTGGACCTAATAGACATCTACAGAACTCTCCACCCCAAATCAACAGAATATATATTTTTTTCAGCACCACACCACAC

CTATTCCAAAATTGACCACATACTTGGAAGTAAAGCTAACCTCAGCAAAAGGAAAAGAACAGAAATGAT|GAC
```
